# Ketorolac Tromethamine Spray Prevents Postendotracheal-Intubation-Induced Sore Throat after General Anesthesia

**DOI:** 10.1155/2016/4582439

**Published:** 2016-11-29

**Authors:** H. L. Yang, F. C. Liu, S. C. Tsai, P. K. Tsay, H. T. Lin, H. E. Liu

**Affiliations:** ^1^Department of Nursing, Chang Gung Memorial Hospital, Linkou, Taoyuan, Taiwan; ^2^Graduate Institute of Clinical Medical Sciences, College of Medicine, Chang Gung University, Taoyuan, Taiwan; ^3^Department of Anesthesiology, Chang Gung Memorial Hospital, Linkou, Taoyuan, Taiwan; ^4^College of Medicine, Chang Gung University, Taoyuan, Taiwan; ^5^Department of Public Health and Center of Biostatistics, College of Medicine, Chang Gung University, Taoyuan, Taiwan; ^6^Department of Rheumatology, Chang Gung Memorial Hospital, Linkou, Taoyuan, Taiwan; ^7^Department of Nursing, College of Nursing, Chang Gung University of Science and Technology, Taoyuan, Taiwan

## Abstract

*Background*. Postoperative sore throat is one of the major complaints of general anesthesia in the postanesthesia care unit. This prospective study investigated the preventive effect of ketorolac tromethamine spray in postendotracheal-intubation-induced sore throat after general anesthesia.* Methods*. Surgical patients undergoing general anesthesia with endotracheal intubation were recruited from a medical center. Patients were randomly assigned to group K (treated with 5% ketorolac tromethamine spray) or group D (treated with distilled water spray). Before intubation, each endotracheal tube was sprayed with the appropriate solution by physicians over the 20 cm length of the cuff. Each group comprised 95 patients fitting the inclusion and exclusion criteria for whom complete data sets were collected. The intensity of the sore throat was measured at 1, 3, 6, and 24 h after surgery, and data were compared.* Results*. The two groups had similar characteristics. Postoperative sore throat was significantly less frequent in group K than in group D (*p* < 0.001) and the pain intensity was significantly lower in group K than in group D at each time point (all *p* < 0.001).* Conclusions*. This study demonstrated that preanesthesia 5% ketorolac tromethamine spray could effectively decrease postendotracheal-intubation-induced sore throat in patients undergoing general anesthesia.

## 1. Introduction

Postoperative sore throat (POST) is one of the major complaints in the postanesthesia care unit [1]. The incidence of POST ranges from 40% to 85.7% [2–4]. Endotracheal intubation or endotracheal cuff pressure could damage the pharyngeal mucosa and lead to postoperative pharyngeal injury [5, 6]. A meta-analysis has revealed that the use of a smaller endotracheal tube (ETT) size can reduce the incidence of POST [7]. Several factors, such as rough handling during intubation, suction, or postendotracheal intubation, can increase the likelihood of developing POST [8]. In addition, female sex, age below 65 years, smoking, higher American Society of Anesthesiology (ASA) physical status, longer duration of anesthesia, and head and neck surgery increase the chances of developing POST [9].

Ketorolac tromethamine can alleviate POST. Ketorolac tromethamine is a racemic mixture, in which the S(−) isomer has strong pharmacological activity. It is a prostaglandin synthesis inhibitor and is an antipyretic nonsteroidal anti-inflammatory drug (NSAID), which is rapidly effective against pain and inflammation [10, 11]. After about 30 min, ketorolac injection becomes effective; its efficacy peaks by 1-2 h, and its effect lasts 4–6 h, with a blood protein-binding rate of about 99%. The drug is mainly metabolized by the liver and excreted by the kidneys and has a half-life of 5-6 h [12, 13]. Locally, a low dose can act via the mucosal membrane to achieve maximum therapeutic effect [14].

In this study, we assessed whether ketorolac tromethamine spray was effective for reducing the symptoms of POST.

## 2. Materials and Methods

### 2.1. Design, Setting, and Sample

This prospective study was approved by the Linkou Chang Gung Memorial Hospital (IRB number 103-6531C) and was registered in ClinicalTrials.gov PRS (registration number NCT02608788). With power set at 80% and a significance level of  0.05 for a two-group design, the G-Power analysis estimated that 95 subjects would be required per group.

The inclusion criteria were as follows: American Society of Anesthesiology (ASA) physical status I–ΙII, age 20–85 years, and undergoing abdominal or orthopedic surgery, which was elective. Exclusion criteria were as follows: history of allergy to NSAIDs, kidney disease, renal dysfunction, peptic ulcer, pregnancy or lactation, surgical site located around the mouth, throat, or neck, use of patient-controlled analgesia (PCA), presence of a nasal gastric tube, difficult airway, or no extubation postoperatively.

Anesthesia was induced intravenously in all subjects, using fentanyl 1.5 *μ*g/kg body weight, propofol 2 mg/kg body weight, and rocuronium 0.6 mg/kg body weight; anesthesia was maintained with sevoflurane 1.5%–2.0%.

**Figure 1:**
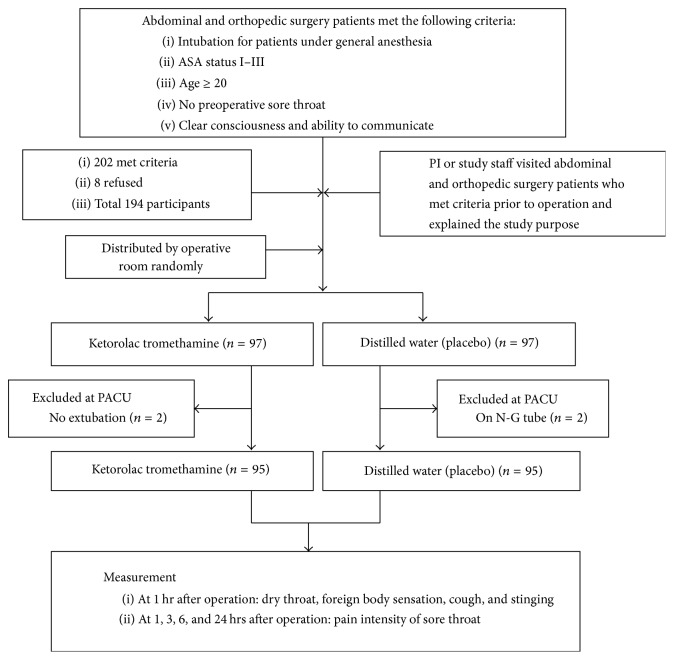
Study flowchart. Flowchart of patients selection and study design: 97 experimental cases and 97 control cases are included.

In the hospital, all patients scheduled for abdominal and orthopedic surgery were admitted 1 day prior to the operation. Patients undergoing such elective surgery from September 2014 to July 2015 were targeted. One researcher listed all potential candidates and considered them against the inclusion criteria. The same researcher visited each potential candidate and explained the purpose of the study and obtained written informed consent. Neither the patients nor the physicians were blinded. The study flowchart is shown in Figure 1.

Each operative room was randomly assigned to either of the two groups and all patients operated on in the same room were treated with the same protocol. In each of the operating rooms, the spray bottles were used to apply the same volume of the different reagents; thus, the anesthesiologists did not know to which group the patients belonged.

*Group K (Treated with 5% Ketorolac Tromethamine Spray)*. Before intubation, each ETT cuff was sprayed 10 times with 8.0 mg (5 cc) of ketorolac tromethamine solution by physicians, covering an area of 20 cm long part of the cuff.

*Group D (Treated with Distilled Water Spray)*. Before intubation, each ETT cuff was sprayed 10 times with 5 cc of distilled water by physicians, covering a 20 cm long part of the cuff.

### 2.2. Measurement and Devices

Research tools, including a pain scale (numeric rating scale [NRS]: 0–10), types of sprays (5% ketorolac tromethamine spray and distilled water spray), and the case data recording sheet, were employed. In 2011, Hjermstad et al. [15] compared three scales for assessing pain intensity, namely, the NRS, verbal rating scale, and visual analogue scales, and found that the NRS was suitable for assessment of pain intensity in adults, with reliability (*r* = 0.95, resp.) and validity correlations ranging from 0.86 to 0.95 [16]. Thus, in this study, the intensity of sore throat was measured using the NRS (0–10 points, with 0 indicating no pain and 10 indicating the worst pain imaginable) at 1, 3, 6, and 24 h after operation.

The case data recording sheet was designed to record the side effects and adverse reactions in patients after surgery and the observed changes in the degree of patient medication for sore throat. This sheet was prepared by researchers from the recording unit and comprised a platform for collecting basic case information, inclusion and exclusion criteria for the case, medical history, surgical anesthetic used, recovery trial drug, intensity of sore throat pain at 1, 3, 6, and 24 h after surgery, side effects and adverse reactions, and other received projects.

### 2.3. Data Analysis

The collected information was analyzed using SPSS for Windows (version 21.0) statistical software package, with the significance level set at *p* < 0.05. Both descriptive and inferential statistics were generated. Sample homogeneity among the two groups was verified by independent *t*-tests and chi-square tests. GEE was selected to compare the throat pain at the four time points of assessment.

## 3. Results

A total of 202 abdominal and orthopedic surgery patients met the inclusion criteria. Eight patients, fearing side effects, refused to participate, while 194 patients agreed to participate in the study. In the PACU (postanesthesia care unit), four patients were excluded; these included two patients on a nasal gastric tube and two patients who were not extubated. Finally, 190 patients were included in these two groups by place of operation. Patients were distributed into two groups, each with 95 patients. The clinical characteristics of the two groups are shown in Table 1. There were no significant differences between the two groups in terms of gender, age, education, presence of a partner, BMI, ASA status, ETT size, or surgical site (*p* > 0.05). Both groups had a similar duration of anesthesia (173.95 ± 63.65 versus 166.63 ± 66.51, *p* > 0.05; Table 1).

**Figure 2:**
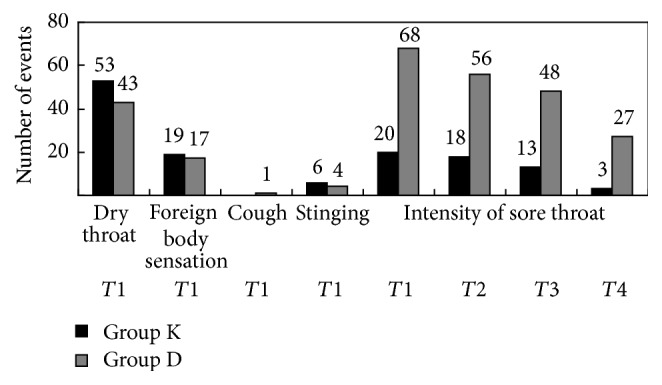
Occurrence and intensity of postoperative adverse events. *T*1: postoperative 1 h; *T*2: postoperative 3 h; *T*3: postoperative 6 h; *T*4: postoperative 24 h. Ratio comparison of postoperative adverse events between ketorolac group and distilled water group.

The postoperative adverse events of patients in the two groups are shown in Table 2. Group K reported a higher frequency of postoperative adverse events than group D, although the differences between the groups were not significant. Both groups had a similar frequency of dry throat (55.8% versus 45.3%, *p* > 0.05), foreign body sensation (20.0% versus 17.9%, *p* > 0.05), cough (0.0% versus 1.1%, *p* > 0.05), and stinging (6.3% versus 4.2%, *p* > 0.05; Figure 2). In group K, 21.1% of the participants experienced sore throat, versus 71.6% in group D. The incidence of postoperative sore throat was significantly lower in group K than in group D at 1 h, 3 h, 6 h, and 24 h postoperatively (*p* < 0.01; Figure 2).

**Figure 3:**
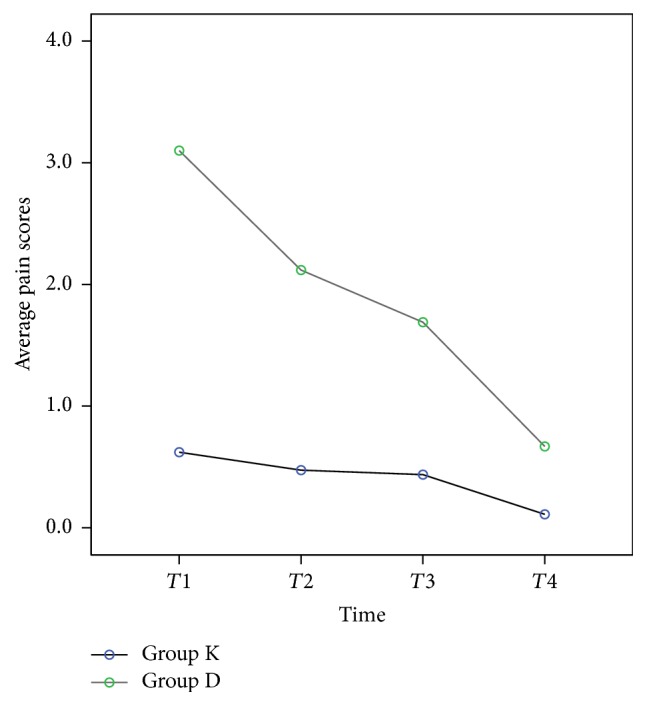
Pain score of postoperative sore throat. Group K was treated with 5% ketorolac tromethamine spray, and group D was treated with distilled water spray. *T*1: postoperative 1 h; *T*2: postoperative 3 h; *T*3: postoperative 6 h; *T*4: postoperative 24 h.

The postoperative intensity of sore throat over time is shown for the two groups in Table 2. GEE showed that patients in group K reported a lesser intensity of sore throat than patients in group D (*p* < 0.01; Table 2). The intensity of postoperative sore throat was significantly lower in group K than in group D at each point of assessment (*p* < 0.001). The intensity of sore throat decreased over time postoperatively, with the worst pain at 1 h after surgery and the least at 24 h after surgery (Table 2; Figure 3).

In this study, none of the 95 patients using 5% ketorolac tromethamine showed any drug allergies or other adverse drug reactions.

## 4. Discussion

Clinical studies are actively exploring safe and effective solutions for sore throat after surgery. This study confirmed that postoperative sore throat is a common problem in postoperative patients as shown in the literature [17, 18].

This study demonstrated that the use of 5% ketorolac tromethamine spray before intubation could reduce the intensity of sore throat and effectively alleviate postendotracheal-intubation-induced sore throat after general anesthesia. We found that the intensity of sore throat in patients treated with 5% ketorolac tromethamine spray was significantly reduced. Previously, 5% ketorolac tromethamine has been shown to be absorbed locally and to be effective for the treatment of pain [14]. NSAIDs have been proven to be effective in treating the incidence and intensity of postoperative sore throat; the intensity of the postoperative sore throat decreased with time, was notably less by 1 h after surgery, and was essentially negligible at 24 h after surgery [19].

In a randomized study (*n* = 20) of endodontic treatment by intranasal ketorolac for endodontic pain, the absorption of the drug via the mucosa offered an effective treatment for endodontic pain from baseline (the first nasal spray) over the 24-hour follow-up period [20]. Ketorolac absorbed through the nasal mucosa was well tolerated and an analgesic effect was achieved within 20 min, without the side effects of morphine-like painkillers. Ketorolac relieves pain through inhibition of arachidonic acid synthesis at the level of cyclooxygenase and has no central opioid effects, making it relatively safe for clinical use [21]. In this study, none of the 95 patients treated with 5% ketorolac tromethamine had any drug allergies or other adverse drug reactions. Previous studies have shown that low doses of ketorolac tromethamine absorbed via the mucosa can effectively relieve pain, without causing the general systemic side effects of analgesics, and are both safe and effective [14, 22].

The effect of ketorolac tromethamine spray on preventing postanesthesia sore throat has not been studied in patients who have undergone intubation for surgery. The present study demonstrated that 5% ketorolac tromethamine spray could reduce the incidence of sore throat in patients undergoing general anesthesia.

The study was limited in that physiological responses after extubation, such as cough, vomiting, and other related factors, were not observed in patients after surgery; however, in future studies, such physiological responses should be investigated. This study was limited to the effects on patients intubated for surgery and general anesthesia; in future, this could be expanded to other populations of patients, for example, for wound pain in patients undergoing oral surgery.

## 5. Conclusion

The results of this study showed that 5% ketorolac tromethamine spray, administered before intubation of the patient for anesthesia, that is, prophylactic administration, could effectively improve patients' postoperative sore throat. None of the patients using 5% ketorolac tromethamine showed any drug allergies or adverse reactions. Therefore, prophylactic use of 5% ketorolac tromethamine may improve sore throat in patients undergoing anesthesia for surgery.

## Figures and Tables

**Table 1:** Clinical characteristics of patients.

	Group K	Group D	*p* value
	*n* = 95	*n* = 95	
	Mean ± SD/%	Mean ± SD/%	
Male/female	38.9/61.1	41.1/58.9	0.769
Age (years)	53.66 ± 15.57	51.09 ± 15.80	0.260
The following junior/above senior middle school	44.2/55.8	40/60	0.559
With partner/without partner	82.1/17.9	73.7/26.3	0.164
Smoker/nonsmoker	13.7/86.3	12.6/87.4	1.000
BMI	25.17 ± 4.05	24.51 ± 4.45	0.341
ASA status I/II/III (%)	9.5/56.8/33.7	3.2/56.8/40	0.136
Endotracheal tube size 6.5/7.0/7.5/8.0 (%)	12.6/52.6/34.7/0	15.8/48.4/34.7/1.1	0.916
Surgical site: abdominal/orthopedic (%)	71.6/28.4	76.8/23.2	0.361
Duration of anesthesia (min)	173.95 ± 63.65	166.63 ± 66.51	0.440

Data are presented as mean ± SD and number (percentage).

BMI: body mass index.

**Table 2:** Generalized estimating equation (GEE) analysis of the postoperative sore throat and different time points.

Variables	*β*-Estimate	SE	95% CI	*p* value
Intercept	0.668	0.1331	0.408–0.929	0.990
Group K	−0.558	0.1529	−0.858–0.258	<0.001
Group D	0^a^			
*T*1	2.432	0.2115	2.017–2.846	<0.001
*T*2	1.449	0.1625	1.131–1.768	<0.001
*T*3	1.021	0.1321	0.762–1.280	<0.001
*T*4	0^a^			

^a^Parameter that statistically represents zero.

Group K was treated with 5% ketorolac tromethamine spray; group D was treated with distilled water spray. *T*1: postoperative 1 h; *T*2: postoperative 3 h; *T*3: postoperative 6 h; *T*4: postoperative 24 h.
